# Utilising alternative cystoscopic schedules to minimise cost and patient burden after trimodality therapy for muscle‐invasive bladder cancer

**DOI:** 10.1002/cam4.5840

**Published:** 2023-03-25

**Authors:** Rahul Krishnatry, Priyamvada Maitre, Anuj Kumar, Tejshri Telkhade, Ganesh Bakshi, Gagan Prakash, Mahendra Pal, Amit Joshi, Santosh Menon, Vedang Murthy

**Affiliations:** ^1^ Department of Radiation Oncology Tata Memorial Centre and Advanced Centre for Treatment, Research and Education in Cancer (ACTREC), Homi Bhabha National Institute (HBNI) Mumbai India; ^2^ Department of Surgical Oncology Tata Memorial Centre and Advanced Centre for Treatment, Research and Education in Cancer (ACTREC), Homi Bhabha National Institute (HBNI) Mumbai India; ^3^ Department of Medical Oncology Tata Memorial Centre and Advanced Centre for Treatment, Research and Education in Cancer (ACTREC), Homi Bhabha National Institute (HBNI) Mumbai India; ^4^ Department of Pathology Tata Memorial Centre and Advanced Centre for Treatment, Research and Education in Cancer (ACTREC), Homi Bhabha National Institute (HBNI) Mumbai India

**Keywords:** cost reduction, cystoscopy, muscle‐invasive bladder cancer, surveillance, trimodality therapy

## Abstract

**Background:**

To assess urinary symptoms and urine cytology as screening tools for cystoscopic detection of local recurrence after bladder‐preserving trimodality treatment (TMT).

**Methods:**

Patients with muscle‐invasive bladder cancer receiving definitive TMT follow‐up three monthly for 2 years, six monthly for the next 3 years and then yearly, with a clinical review, urine cytology and cystoscopy at each visit (triple assessment, TA). Grade 2+ cystitis/haematuria absent/present was scored 0/1, and urine cytology reported negative/suspicious or positive was scored 0/1, respectively. The performance of these two parameters for predicting local recurrence in cystoscopic biopsy was tested. Other hypothetical surveillance schedules included cystoscopy on alternate visits (COAV), or suspected recurrence (COSR), six‐monthly COSR and six‐monthly TA.

**Results:**

A total of 630 follow‐up visits in 112 patients with 19 recurrences (7 muscle invasive, 12 non‐muscle invasive) at a median follow‐up of 19 months were analysed. The sensitivity and specificity of clinical symptoms were 47.4% and 92%, and for urine cytology 58% and 85%, respectively. The combination of clinical symptoms and cytology (COSR) was 95% sensitive and 78% specific for local recurrence but 100% sensitive for muscle‐invasive recurrence. Both COAV and COSV schedules showed a high area under the curve (AUC) for detecting local recurrence (COAV = 0.84, COSR = 0.83), muscle‐invasive recurrence (AUC = 0.848 each) and non‐muscle‐invasive recurrence (COAV = 0.82, COSR = 0.81); reducing the need for TAs by 64% and 67% respectively, and overall cost by 18% and 33%, respectively.

**Conclusion:**

Cystoscopy at suspected recurrence during follow‐up is safe and the most cost‐effective for detecting muscle‐invasive local recurrences, while cystoscopy at alternate visits may be more optimal for detecting any local recurrence.

## Graphical abstract

We try optimising the need for cystoscopy at every visit on post‐trimodality treatment follow‐up.

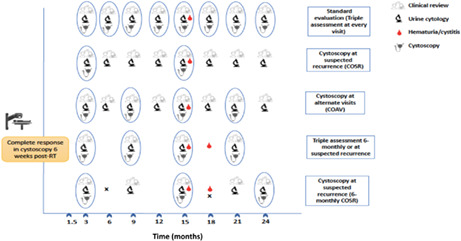

## 1 INTRODUCTION

Bladder preservation using a multi‐modality approach is a standard alternative to radical cystectomy for treating muscle‐invasive bladder cancer (MIBC) in selected patients.^1^, ^2^ Tri‐modality therapy (TMT) is the most robust method of sparing the native bladder, consisting of maximal transurethral resection of the bladder tumour (TURBT) followed by external beam radiation therapy and concurrent radio‐sensitising chemotherapy.^3^ Post TMT, triple assessment (TA) with regular cystoscopic surveillance in addition to clinical review and urine cytology is recommended. There is a high risk of non‐muscle‐invasive and muscle‐invasive recurrences in the urinary tract.^1^, ^4^ The frequency and intensity of follow‐up suggested are based on consensus or intuition than any evidence in the literature.

Clinically, symptoms such as haematuria, urinary frequency, urgency and dysuria at follow‐up may herald a local recurrence. However, these are non‐specific on their own and are often sequelae of TMT.^5^ Given the high risk of non‐muscle‐invasive and muscle‐invasive local recurrences, major guidelines recommend close surveillance post‐TMT with urine cytology and cystoscopy every 3–6 months.^1^, ^2^, ^4^ For example, NICE guidelines recommend rigid or flexible cystoscopy three‐monthly for 2 years, while EAU suggests three‐ to four‐monthly scopies for the initial 3 years.^1^, ^6^, ^7^

Urine cytology identifies malignant cells exfoliated from the urothelium into the urine. It is inexpensive, non‐muscle invasive and has high specificity for detecting high‐grade recurrence but rather low sensitivity, especially for lower grade recurrences.^8^ Cystoscopy with histopathological confirmation of visible suspicious lesions is the gold standard for the diagnosis of local recurrence. However, the optimal frequency of surveillance cystoscopy post‐TMT is not known, and such an invasive procedure at every follow‐up is a significant burden on the patients and healthcare systems. Hence, this retrospective study assessed the combination of urinary symptoms and urine cytology as a screening tool to guide the optimal frequency of cystoscopy for detecting local recurrence after bladder‐sparing TMT.

## 2 METHODS AND MATERIALS

### 2.1 Study population

Clinical records of patients with biopsy‐proven non‐metastatic MIBC who underwent bladder preservation with radical intent TMT from 2009 to 2019 at our institute were retrospectively evaluated. Patients with an incomplete response at first follow‐up or those treated with palliative radiotherapy were excluded from this study. This retrospective study was approved by the institutional review board (IEC no. 900750).

### 2.2 Study procedures

Bladder‐preserving TMT consisted of maximal TURBT followed by chemoradiation. All patients were treated with an adaptive ‘plan of the day’ technique using an intensity‐modulated technique under daily image guidance.^9^ The total dose of 64 Gy in 32 fractions over 6 weeks was prescribed to the entire bladder, with or without simultaneous integrated boost up to 68 Gy to the gross tumour and 55 Gy to the pelvic lymph nodes. Concurrent chemotherapy included either cisplatin 30 mg/m^2^ or gemcitabine 75–100 mg/m^2^ given once weekly. As per the institutional protocol, follow‐up post‐TMT was three‐monthly for the first 2 years, six‐monthly for the next 3 years, and yearly after that. A TA consisting of clinical examination, urine cytology and cystoscopy was performed at every follow‐up. Cystoscopy was performed under local anaesthesia and post‐therapy cystitis or visible lesions were documented.

Further biopsy from suspicious areas was performed under general anaesthesia if clinically indicated. Surveillance cross‐sectional imaging was not routinely performed unless clinically suspected of a nodal or distant progression. For this study, details of clinical review, cytology and cystoscopy at each follow‐up visit were collected till the last recorded follow‐up or first confirmation of local recurrence. After identifying a recurrence, patients were censored from the study.

Urinary symptoms of haematuria (gross bleeding through the urinary tract) and cystitis (urgency, frequency and dysuria) were graded as per Common Terminology Criteria for Adverse Events version 5.0. Cystitis of Grade 2 or higher and visible haematuria were considered ‘positive’ symptoms. Urine cytology was categorised as either negative or suspicious (atypical or positive) for malignancy. Cystoscopy‐directed histopathological confirmation of non‐muscle‐invasive or muscle‐invasive tumours was considered the gold standard for local recurrence.

### 2.3 Alternative schedule selection

**FIGURE 1:**
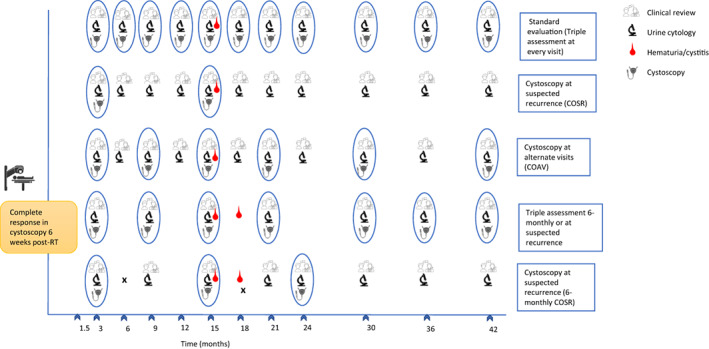
Standard triple assessment followed for all patients and alternative hypothesised four schedules.

After completion of planned chemoradiation, urine cytology and cystoscopy are performed at the first follow‐up visit at 6–8 weeks to document the complete response of the tumour. To evaluate the possibility of avoiding cystoscopy at every visit or frequency of visits, alternative schedules were proposed. Alternative schedules were hypothesised within a multidisciplinary discussion by the uro‐oncology team (two radiation oncologists, three urosurgeons, three medical oncologists and one uropathologist), focusing on feasibility and meaningful cost reduction without increasing risk of missing local recurrences. We constructed 10 alternative surveillance strategies by varying cystoscopy frequency, cytology and clinical symptom assessment. Four of these were selected based on 2 × 2 prioritisation matrix among the multidisciplinary member group (Figure 1). Two of the alternatives were to either perform a clinical review and/or cytology at every visit, or reserve cystoscopy only if recurrence is suspected based on clinical review and cytology (cystoscopy on suspected recurrence, COSR); or secondly comprised of a clinical review and cytology at every visit combined with cystoscopy at alternate follow‐up visits (cystoscopy on alternate visit, COAV); on remaining alternate visits, COSR rules were applied for cystoscopy. Two other alternatives accepted were to propose six‐monthly TAs for the first 5 years and then yearly thereafter (6mTA), and the last one as six‐monthly follow‐up for the first 5 years and yearly thereafter but cystoscopy only when recurrence is suspected on clinical review and/or cytology (6m‐COSR).

### 2.4 Statistical analysis

Descriptive statistics were used to summarise all patient‐related parameters. Sensitivity, specificity, positive predictive value, negative predictive value (NPV) and accuracy were calculated for clinical symptoms, cytology and their combinations. The alternative schedules with >70% sensitivity for local recurrence were only considered for detailed analysis. The sensitivity and specificity of these two schedules were calculated to compare with the present practice of TA at every visit.

Receiver operating characteristic (ROC) statistics were used to analyse the performance of the combination of clinical review and urine cytology for diagnosing tumour recurrence. The absence or presence of ‘positive’ bladder symptoms as defined above was scored as ‘0’ and ‘1’, respectively. Urine cytology reported as negative/suspicious was scored as 0/1, respectively. The sum of these two scores was used to generate the ROC curve to predict recurrent disease as diagnosed in the cystoscopic biopsy. Statistical Package for the Social Sciences (SPSS) software, version 22.0 (IBM Inc.) was used to do ROC analysis.

### 2.5 Cost analysis

If the direct typical cost to the patient for a TA follow‐up visit at our institution (grant‐in‐aid government institute) is considered as 1 unit, it was estimated that the cost of cystoscopy under local anaesthesia would be 0.5 units. Based on this, the cost for various alternative schedules was estimated in our cohort, identifying the saving associated with each schedule. The cost sparing for alternate schedules as compared to standard schedules was estimated in percentage. This was estimated as a percentage difference from the standard schedule (presumed as 100%).

## 3 RESULTS

A total of 630 follow‐up visits by 112 patients with the complete response at first follow‐up after bladder‐preserving TMT were included in this study. Patient characteristics are detailed in Table 1. Neoadjuvant chemotherapy was given in 23 (21%) patients, most commonly gemcitabine combined with cisplatin or carboplatin. Concurrent chemotherapy was administered in 81 (72%) patients, most commonly platinum‐based (63%), followed by gemcitabine (35%). The median follow‐up duration was 19 months. Twenty patients (17.9%) defaulted their TAs overall at a median follow‐up of 9 months (range: 3–82 months). A mean ± SD of 3.5 ± 7.3 weeks' delay noted for scheduled 630 TA visits which were committed. Cystoscopy was suspicious in 29 visits, of which subsequent biopsy was performed in 27 visits (two patients did not report for the scheduled biopsy). Histopathological recurrence was confirmed in 19 out of the 27 samples (70.4%), of which 7 were muscle‐invasive recurrences and 12 were non‐muscle‐invasive recurrences. Of the 12 non‐muscle‐invasive recurrences, 10 (83.3%) were of high grade. Three‐year distant recurrence rate was 16% in this cohort, and no patient had isolated pelvic nodal relapse.^9^

**TABLE 1:** Patient characteristics.

Characteristic	*N* = 112 (%)
Age (years)	
Median (interquartile range)	65(57.5–72.75)
≤65	61 (54.4)
>65	51 (45.6)
Gender	
Male	104 (92.8)
Female	08 (7.2)
Comorbidities	
No	61 (54.4)
Yes	51 (45.6)
Histological subtypes	
Urothelial	96(85.7%)
Squamous focal	8 (7.15%)
Small cell/neuroendocrine/sarcomatoid	8 (7.15%)
T stage	
T1	11 (9.8)
T2	67 (59.8)
T3	26 (23.2)
T4	06 (5.2)
N stage	
N0	109 (97.3)
N1	01 (0.8)
N2	00
N3	01 (0.8)
Neoadjuvant chemotherapy	
Yes	23 (20.5)
No	89 (79.5)
Concurrent chemotherapy	
Yes	81 (72.3)
No	31 (27.7)

The estimated performance of clinical symptoms, cytology, and their combinations as a screening tool to detect muscle‐invasive and non‐muscle‐invasive recurrences are detailed in Table 2. Malignant cells on cytology had a low sensitivity of 26.3% (5/19) for local recurrence but was highly specific (96.1%; 587/611). Including the presence of atypical cells in urine cytology within the screening criteria increased sensitivity to 57.9% (11/19) but reduced specificity to 85% (519/611). The presence of clinical symptoms relating to Grade 2 cystitis or haematuria as screening criteria yielded a sensitivity of 47.4% (9/19) and specificity of 92% (562/611). The highest sensitivity was observed when the screening criteria included a combination of clinical symptoms and cytology (COSR). In the presence of either Grade 2 cystitis or haematuria or cytology being atypical or positive, sensitivity increased to 95% (18/19) for local recurrence overall. This criterion was 100% sensitive for detecting muscle‐invasive local recurrence.

**TABLE 2:** Screening performance of clinical symptoms and cytology to detect local recurrence.

Screening criteria	Type of local recurrence	Sensitivity % (numerator/denominator)	Specificity % (numerator/denominator)	PPV % (numerator/denominator)	NPV % (numerator/denominator)	Accuracy % (numerator/denominator)
Cytology positive	Any	26.3% (5/19)	**96.1% (587/611)**	17.2% (5/29)	**97.7% (587/601)**	**94% (592/630)**
	Non‐muscle invasive	25% (3/12)	**95.8 (592/618)**	10.3% (3/29)	**98.5% (592/601)**	**94.4% (595/630)**
	Muscle Invasive	28.6% (2/7)	**95.7% (596/623)**	6.9% (2/29)	**99.2% (596/601)**	**94.9 (598/630)**
Cytology atypical or positive	Any	57.9% (11/19)	84.9% (519/611)	10.7% (11/103)	**98.5% (519/527)**	84.1% (530/630)
	Non‐muscle invasive	58.3% (7/12)	84.5% (522/618)	6.8% (7/103)	**99.1% (522/527)**	84% (529/630)
	Muscle invasive	57.1% (4/7)	84.1% (524/623)	3.9% (4/103)	**99.4% (524/527)**	83.8% (528/630)
Haematuria (Grade 2+)	Any	42.1% (8/19)	**92.8% (567/611)**	15.4% (8/52)	**98.1% (567/578)**	**91.3% (575/630)**
	Non‐muscle invasive	33.3% (4/12)	**92.2% (570/618)**	7.7% (4/52)	**98.6**% (**570/578**)	**91.1% (574/630)**
	Muscle invasive	57.1% (4/7)	**92.3% (575/623)**	7.7% (4/52)	**99.5**% (575/578)	**91.9% (579/630)**
Cystitis (Grade 2+)	Any	15.8% (3/19)	**98.7% (603/611)**	27.3% (3/11)	**97.4**% (603/619)	**96.2% (606/630)**
	Non‐muscle invasive	16.7% (2/12)	**98.5% (609/618)**	18.2% (2/11)	**98.4**% (609/619)	**97% (611/630)**
	Muscle invasive	14.3% (1/7)	**98.4% (613/623)**	9.1% (1/11)	**99**% (613/619)	**97.5% (614/630)**
Either cystitis or haematuria	Any	47.4% (9/19)	**92% (562/611)**	15.5% (9/58)	**98.3**% (562/572)	**90.6**% (571/630)
	Non‐muscle invasive	41.7% (5/12)	**91.4% (565/618)**	8.6% (5/58)	**98.8**% (565/572)	**90.5% (570/630)**
	Muscle invasive	57.1% (4/7)	**91.3% (569/623)**	6.9% (4/58)	**99.5**% (569/572)	**91% (573/630)**
Cystitis (Grade 2+) or haematuria (Grade 2+) or cytology atypical/ positive (COSR)	Any	**94.7**% (18/19)	78.2% (478/611)	11.9% (18/151)	**99.8**% (478/479)	78.7% (496/630)
	Non‐muscle invasive	**91.7**% (11/12)	77.4% (478/618)	7.3% (11/151)	**99.8**% (478/479)	77.6% (489/630)
	Muscle invasive	**100**% (7/7)	76.9% (479/623)	4.6% (7/151)	**100**% (479/479)	77.1% (486/630)

The bold values indicate specificity >90%.

Abbreviations: COSR, cystoscopy on suspected recurrence; PPV, positive predictive value; NPV, negative predictive value.

**FIGURE 2:**
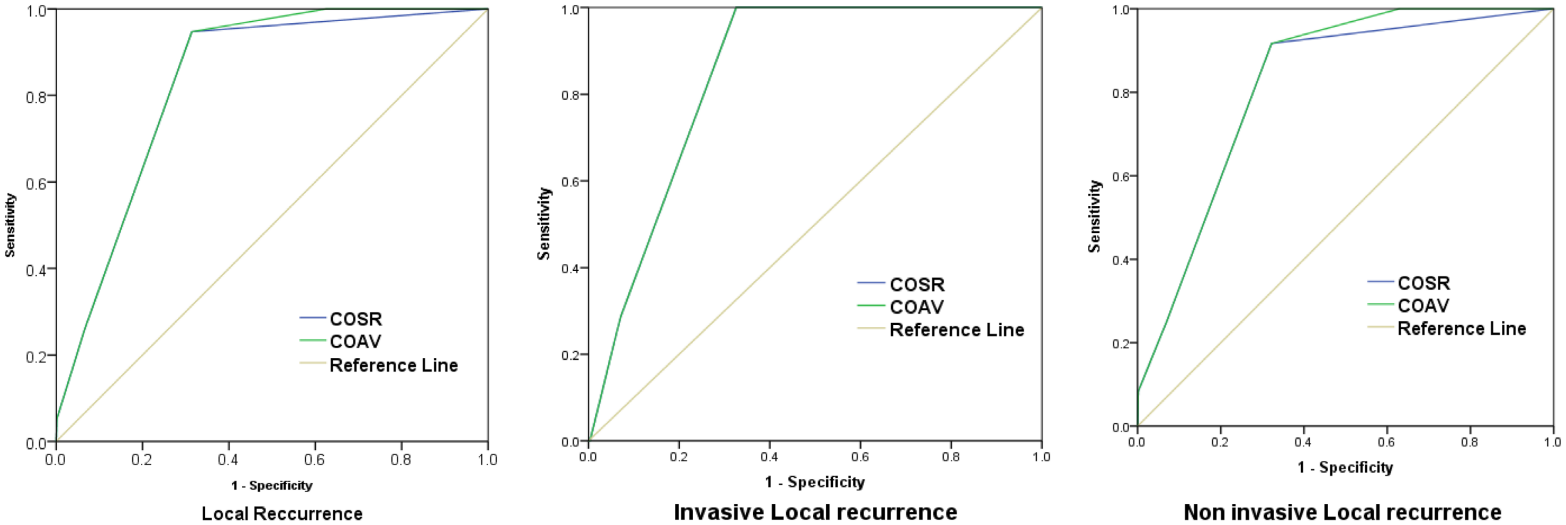
Receiver operating characteristic (ROC) for cystoscopy on suspected recurrence (COSR), and cystoscopy on the alternate visit (COAV) schedules for all local, muscle‐invasive and non‐muscle‐invasive recurrences.

Among the alternative schedules, the 6mTA and 6m‐COSR, each would have missed 42.9% (8/19) of all muscle‐invasive recurrences and 42.1% (3/7) of all local recurrences. Given the unacceptable rates of misses, these schedules were not considered for further analysis. The sensitivity and specificity of the other alternative assessment schedules are shown in Table 3. COSR schedule showed high sensitivity of 95% (18/19) and specificity of 78.2% (478/611) for the detection of local recurrences. While sensitivity was somewhat lower at 92% (11/12) for non‐muscle‐invasive recurrences, it increased to 100% for the detection of muscle‐invasive local recurrences. COAV schedule showed 100% sensitivity and lower specificity of 47.3% (289/611) for local recurrence. It was also 100% sensitive for detecting muscle‐invasive recurrence, with a specificity of 46.4% (289/623). Both schedules showed high AUC values for detecting local recurrence, being 0.828 for COSR and 0.838 for COAV. For muscle‐invasive recurrence, AUC was 0.848 for both schedules, while for non‐muscle‐invasive recurrence, AUC was 0.808 for COSR and 0.824 for the COAV schedule (Figure 2).

**TABLE 3:** Screening performance of alternative assessment schedules.

Type of local recurrence		Alternative assessment schedule	
		Cystoscopy on suspected recurrence (numerator/denominator)	Cystoscopy on alternate visits (numerator/denominator)
Any recurrence	Sensitivity % (numerator/denominator)	94.7% (18/19)	100% (18/18)
	Specificity % (numerator/denominator)	78.2% (478/611)	47.3% (289/611)
Non‐invasive	Sensitivity % (numerator/denominator)	91.7% (11/12)	100% (12/12)
	Specificity % (numerator/denominator)	77.4% (478/618)	46.8% (289/618)
Invasive	Sensitivity % (numerator/denominator)	100% (7/7)	100% (7/7)
	Specificity % (numerator/denominator)	76.9% (479/623)	46.4% (289/623)

**FIGURE 3:**
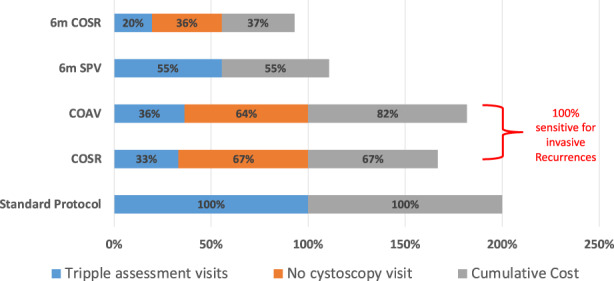
Relation between cost and type of assessments in various schedules. The left half of the bar for each follow‐up method shows the number of triple assessment (TA) visits versus non‐cystoscopy visits are needed compared with standard method (needing 100% TA with cystoscopy at each visit), The right half of the bar shows corresponding percentage decrease in cost of overall surveillance schedule on the population compared with standard schedule (presuming 100% cost). COAV, cystoscopy on the alternate visit; COSR, cystoscopy on suspected recurrence; SPV, standard protocol visit, 6m.

The impact on the number of TAs and percentage change in overall cost as compared to the standard follow‐up schedule is shown in Figure 3. Although the 6m‐COSR and 6mTA would be the most cost economical, the clinical application would not be acceptable due to a significant number of events being missed. Both COSR and COAV would reduce the need for TAs by 67% and 64%, respectively, reducing the cost by 33% and 18%, respectively. Although both schedules timely identified all muscle‐invasive recurrences, COAV with an additional 15% cost would identify one non‐muscle‐invasive local recurrence which would be missed or delayed with COSR.

## 4 DISCUSSION

The various clinicopathological factors associated with local recurrence after TMT are not clearly defined. Detection of muscle‐invasive local recurrence would invariably lead to salvage cystectomy. Hence factors associated with intact‐bladder disease‐free survival can be considered a surrogate for local recurrence. In a large retrospective cohort, complete response to chemo‐radiation (HR: 0.16, *p* = <0.001), absence of hydronephrosis (*p* = <0.001) and complete TURBT (HR: 0.72, *p* = 0.02) were associated with better intact‐bladder disease‐free survival.^10^ A pooled analysis of various RTOG trials which included patients with MIBC receiving bladder‐preserving combined modality therapy, reported muscle‐invasive local recurrences within 5 and 10 years as 13% and 14%, respectively, while non‐muscle‐invasive recurrences were 31% and 36%, respectively.^11^ The incidence of muscle‐invasive recurrence is quite low after the first 5 years of follow‐up. In our previously published patient cohort with although a shorter follow‐up, we had noted similar local reoccurrence rates.^9^ In the current study cohort, we had excluded patients with an incomplete response at first assessment post‐TMT and also, patients were censored post‐first recurrence; the overall local recurrence rates were in the expected range.

For early detection of local recurrence and timely salvage, various guidelines recommend a stringent protocol for regular assessments with the clinical review, cytology and cystoscopy at every follow‐up. However, various guidelines have varied recommendations. NICE guidelines recommend rigid cystoscopy at 3 months after TMT, followed by rigid or flexible cystoscopy three‐monthly for 2 years, six‐monthly for the next 2 years and yearly thereafter.^6^ According to the EAU, patients undergoing curative‐intent TMT should be followed up every 3–4 months for 3 years initially and 6 months thereafter.^7^ According to consensus guidelines by AUA, ASCO, ASTRO and SUO, follow‐up after TMT must include regular surveillance with CT scans, cystoscopy and urine cytology.^1^ While this schedule of multiple invasive, costly and frequent investigations is not evidence‐based, long‐term intensive cystoscopic surveillance aims to allow timely salvage therapy for local recurrence.

The present study shows that a combination of urinary cytology and clinical review is a highly sensitive tool and hence a good screening test in the follow‐up period, which could potentially avoid the invasive procedure of cystoscopy at every visit. The COSR schedule, that is, cystoscopy triggered by the presence of any of the factors—Grade 2+ haematuria, Grade 2+ cystitis, or urine cytology showing atypical or malignant cells—was 100% sensitive for detecting muscle‐invasive local recurrence, and 92% sensitive for non‐muscle‐invasive recurrence. Restricting cystoscopy to alternate visits (unless triggered by suspicious symptoms or cytology), the COAV schedule also showed 100% sensitivity for detecting muscle‐invasive and non‐muscle‐invasive recurrences. These proposed alternative schedules also showed a significant potential reduction of costs by 33% for COSR and 18% for the COAV schedule. Although this estimated benefit in cost may differ from one healthcare system to another depending on the cost of cystoscopy and associated indirect cost of logistics, overall, it may be a win‐win situation for both patients and the healthcare system. The six‐monthly schedules tested were found to be associated with >40% missed or delayed identification of local recurrences and hence deemed unsuitable.

Generally, a screening tool with good sensitivity and reasonable specificity is considered acceptable. Urine cytology is a simple and non‐invasive investigation which identifies malignant cells exfoliated from the urothelium into the urine and has shown high sensitivity and specificity to detect carcinoma in situ and high‐grade lesions.^12^ Clinically, haematuria is one of the most common symptoms associated with urothelial carcinoma. Other symptoms like frequency, urgency and dysuria can be the presenting symptoms of a local recurrence.^13^ In the present study, a combination of urine cytology and clinical review was observed to give high sensitivity of >90% and reasonable specificity of 60%–70% for the detection of local recurrences, reducing cystoscopy to alternate visits or at suspected recurrence. Compared with a few other screening methods, the sensitivity of mammography for breast cancer has been reported to be as high as 97%, with a specificity of about 65%.^14^ The sensitivity of pap smear for cancer cervix has been observed to be about 75%, with specificity exceeding 90%.^15^ Both the surveillance schedules proposed in the present study have demonstrated similar utility for screening local recurrences.

Cystoscopy‐intensive surveillance protocols incur significant costs to the patient and the healthcare systems. The invasive nature of cystoscopy contributes to patient discomfort and reduces compliance with regular follow‐up visits. It was noted by most of our team that many patients were not comfortable with cystoscopies (rigid more than flexible) and tried to delay/avoid the visits. This inspired us to look at our data to understand whether it is necessary to have such a stringent schedule. Instrumentation increases the risks of urinary tract infections, haematuria, dysuria, injury to the bladder or urethra, and iatrogenic stricture.^16^ These risks in an irradiated bladder should not be considered trivial. These procedural anxiety and/or discomfort have been previously reported in up to two‐thirds of patients.^17^ Cystoscopic evaluation is considered the gold standard for surveillance due to its high sensitivity for detecting recurrence. Still, most of the reported surveillance outcomes for its performance are from low‐grade/non‐MIBC patients.^18^, ^19^ In our cohort, cystoscopy was only 70% specific to detect any recurrence. The risks and benefits of intensive cystoscopy‐based surveillance at every visit in a post‐TMT setting are yet to be evaluated in detail.

Also, post‐TMT long‐term outcomes from various large series suggest that these patients are at much higher risk of distant and nodal metastasis which should probably be more aggressively screened. If identified earlier, systemic therapy salvage may be more effective, especially with recent advances.^20^ The cross‐sectional imaging may further decrease the need for stringent cystoscopies at all visits.

A few limitations of this study are acknowledged. The study cohort is small with relatively fewer local recurrences, though reasonable for such a selected indication in a less common situation. As most local recurrences occur within the first 2 years, a larger sample by pooling multicentric data may increase the robustness of analyses. Inherent shortcomings with retrospective analysis of patients treated outside the clinical trial framework may persist, despite all patients receiving nearly homogenous trimodality treatment at a single institution. Clinical haematuria and other symptoms may not be very rigorously recorded in routine practice. The routine availability of CT pelvis or targeted USS bladder may help improve the specificity of clinical criteria to reduce the need for cystoscopic evaluation. Urine microscopy for occult blood or other urinary biomarkers for bladder cancers may increase the sensitivity.^21^ The impact of a very local recurrence rate in a very different patient population such as old debilitated patients treated with radical radiotherapy alone, cannot be predicted in the current study. The indirect costs of an additional clinic visit for cystoscopy‐guided biopsy, and the cost of missing a recurrence or delayed recognition of a recurrence have not been included in the cost estimates. The present analysis is specific to the non‐profit economic model followed at the treating institute, and this model may lead to a different calculation model depending on the relative cost of clinical visits, cytology and cystoscopy. However, while the magnitude of economic benefit may vary, the present study intends to demonstrate the feasibility of a less invasive surveillance approach towards follow‐up post‐bladder preservation which is economical as well. We expect that with this information, other groups will collaborate and look at its wider validity, leading to guidelines that balance cost/resource utilisation with desired patient safety and TMT success. Till then, patients and physicians can have an informed choice for continuing more stringent protocols.

## 5 CONCLUSION

For patients treated with bladder preservation for MIBC, a combination of clinical symptom review and urinary cytology at every follow‐up, with cystoscopy reduced to suspected recurrence, may be enough to detect most muscle‐invasive recurrences. Additional alternate visits to cystoscopies may be justified at an additional cost and inconvenience to detect most non‐muscle‐invasive recurrences. Prospective evaluation and future testing with additional non‐invasive office tests may be tried to improve the sensitivity and specificity to predict the optimal need for cystoscopy.

## AUTHOR CONTRIBUTIONS

**Rahul Krishnatry:** Conceptualization (lead); data curation (supporting); formal analysis (supporting); investigation (supporting); methodology (lead); project administration (lead); resources (lead); supervision (lead); visualization (equal); writing – original draft (supporting); writing – review and editing (lead). **Priyamvada Maitre:** Conceptualization (supporting); data curation (equal); formal analysis (equal); investigation (equal); methodology (equal); writing – original draft (lead); writing – review and editing (equal). **Anuj Kumar:** Conceptualization (supporting); data curation (equal); formal analysis (equal); investigation (equal); methodology (equal); writing – review and editing (equal). **Tejshri Telkhade:** Data curation (equal); formal analysis (equal); investigation (equal); methodology (equal); writing – original draft (equal). **Ganesh Bakshi:** Conceptualization (supporting); project administration (equal); supervision (equal); writing – review and editing (equal). **Gagan Prakash:** Formal analysis (equal); methodology (equal); project administration (equal); writing – original draft (equal); writing – review and editing (equal). **Mahendra Pal:** Formal analysis (equal); investigation (equal); project administration (equal); supervision (equal); writing – original draft (equal); writing – review and editing (equal). **Amit Joshi:** Formal analysis (equal); investigation (equal); project administration (equal); supervision (equal); writing – original draft (equal); writing – review and editing (equal). **Santosh Menon:** Data curation (equal); formal analysis (equal); investigation (equal); methodology (equal); supervision (equal); writing – original draft (equal); writing – review and editing (equal). **Vedang Murthy:** Conceptualization (supporting); data curation (equal); formal analysis (equal); investigation (equal); methodology (equal); project administration (supporting); supervision (equal); validation (equal); writing – original draft (equal); writing – review and editing (equal).

## CONFLICT OF INTEREST STATEMENT

The authors have no conflict of interest to declare.

## Data Availability

The data that support the findings of this study are available from the corresponding author upon reasonable request.
